# The tower of babel in hematology: The World Health Organization and International Consensus Classification systems

**DOI:** 10.1016/j.htct.2025.103985

**Published:** 2025-09-20

**Authors:** Daniel Mazza Matos, Eduardo Magalhaes Rego

**Affiliations:** a Flow Cytometry Division, Cell Processing Center (CPC), Center of Hematology and Hemotherapy of Ceará (HEMOCE), Brazil; b Faculty of Medicine, University of Fortaleza (UNIFOR), Brazil; c Faculty of Medicine of the University of São Paulo, Department of Clinical Medicine of the Faculty of Medicine of USP, University of São Paulo, Brazil

Let us begin with a thought experiment. Imagine, dear reader, that you and we − the authors − are touring a large zoo. As we pass by the reptile section, Dr. Rego, who to my knowledge knows nothing about reptiles, suddenly exclaims: “Look at this beautiful specimen of an alligator!” Observing the animal, I concur: “Indeed.” However, you, the reader, upon seeing the same animal, object: “This is not an alligator, but a crocodile.” Dr. Rego reexamines the animal and insists: “I am certain this is an alligator. I have seen many alligators in other zoos, and I am confident this is one.” Yet you persist: “It is easy to see that this is not an alligator, but a crocodile; one only needs to examine its features carefully.” The discussion begins to escalate but is abruptly interrupted when our guide announces it is time to move on to the bird section. Later, I find myself reflecting on the nature of classification and what it means to define entities in the world. Was that reptile an alligator or a crocodile? As someone who is not an expert in herpetology, I cannot say with certainty. However, I am sure of one thing: the animal was either an alligator or a crocodile − it could not be both. In philosophical terms, this presents a genuine dichotomy: the reptile in question must be one or the other. These two categories are mutually exclusive. Given that the creature was a single, individual animal, it was either an alligator or, alternatively, a crocodile, but never both.

But what do alligators and crocodiles have to do with issues in hematology? Believe it or not, dear reader, the fact is that nowadays hematologists not only fail to distinguish between alligators and crocodiles (which in itself is not a serious concern, since such distinctions are strictly the domain of zoologists), but also, and this is a real problem, they have come to believe that “the same animal can be both an alligator and a crocodile!” Or, what amounts to the same thing, that a single hematologic disease can be simultaneously classified as disease X and disease Y. Allow us to explain this in a more appropriate way.

The current problems began in 2020, when preparations for the final version of the 5th Edition of the World Health Organization (WHO) Classification − commonly referred to as the “blue book” − were initiated. However, a brief historical overview is necessary to understand the present situation. Up until the 4th Edition of the WHO blue book, a collaborative group of hematologists, pathologists, oncologists, and geneticists formed the Clinical Advisory Committee (CAC), which operated under the auspices of the International Agency for Research on Cancer (IARC), the Society for Hematopathology (SH), and the European Association for Haematopathology (EAHP). Following each CAC meeting, the principal pathologists worked to resolve the difficult issues related to the committee’s recommendations and published their conclusions in scientific articles prior to the formal release of the WHO blue book [1]. However, as reported by Daniel Arber and colleagues [1], “In 2020, Ian Cree, Head of the Evidence Synthesis and Classification Branch of the IARC in charge of the publication of the WHO blue books, notified SH and EAHP that IARC was ending the successful partnership with SH and EAHP for the 5th edition WHO classification of hematopoietic tumors and that they would no longer follow the process described above for the three prior books.” Next, “the Executive Committees of the SH and EAHP organized different multidisciplinary working groups that culminated in the CAC meeting held in Chicago in September 2021.” Finally, the CAC members published the International Consensus Classification (ICC) of Myeloid and Lymphoid Neoplasms in four articles [2, 3, 4, 5]. For its part, the WHO published its independent and final classification in the traditional blue book format in 2024 [6].

At first glance, this dispute between two professional groups may appear to be a remote concern for hematologists who lead demanding clinical lives, often far removed from academic debates and focused on treating patients with life-threatening diseases. And indeed, this may largely be the case. In fact, a recent study comparing the WHO and ICC classifications found that only 1.3 % of acute myeloid leukemia (AML) cases showed “major diagnostic discrepancies” − defined as differences in diagnosis with significant and clear therapeutic implications [7]. However, consider the following scenario: a patient presents with anemia, megakaryocytic dysplasia, 7 % bone marrow blasts, and mutations in *DNMT3A, NRAS*, and *NPM1*. According to the WHO blue book, an “increase in blasts in the peripheral blood and/or bone marrow” is essential for an AML diagnosis. Traditionally, >5 % in bone marrow or >2 % in the blood have been considered abnormal. Thus, according to the WHO, the diagnosis for this case is AML with mutated *NPM1*. In contrast, according to the ICC, because the blast percentage is below 10 %, this patient would be diagnosed with myelodysplastic syndrome, not otherwise specified (MDS-NOS). This discordance between the WHO and ICC regarding the minimum blast percentage for the diagnosis of AML is a very strange situation because, in effect, the patient in question clearly has a single disease, which cannot simultaneously be classified as both AML with mutated *NPM1* and MDS-NOS.

The crucial point here is that the existence of two divergent systems for the classification of AML creates problems that extend beyond the classification of individual cases. For instance, due to the coexistence of the WHO and ICC proposals, the 2024 Brazilian consensus on acute promyelocytic leukemia (APL) does not clearly specify the minimum blast percentage required for an APL diagnosis [8].

There are two potential solutions to this problem. The first is to accept both classifications. The second is to pursue rapid reconciliation between the WHO and ICC. We believe the latter is the best option. Indeed, we recently adopted an unorthodox approach to this issue and concluded that the existing literature already supports a single diagnostic criterion for at least five clinical entities within the group of “AML subcategories with defining genetic abnormalities”: AML with *PML::RARA* rearrangement, AML with *NPM1* mutation, AML with *KMT2A* rearrangement, AML with *MECOM* rearrangement, and AML with in-frame *bZIP CEBPA* (Table 1) [9]. Meanwhile, we believe that the best approach in cases of conflicting diagnoses is to treat patients according to the most appropriate available therapy, guided by the following principles: (1) avoid categorizing an aggressive neoplasm as a “low-grade” disease (for example, diagnosing MDS when AML is the most accurate diagnosis), thereby preventing undertreatment; and (2) avoid categorizing a less aggressive neoplasm as a more aggressive one (such as diagnosing AML when MDS is the most accurate diagnosis), thereby preventing overtreatment [9].

**Table 1:** Preliminary proposal for a unified World Health Organization classification and International Consensus Classification.

AML with defining genetic abnormalities	Blast cutoff
APL with *PML::RARA* FUSION	Increase peripheral blood and/or bone marrow blasts
AML with *NPM1* mutation	
AML with *KMT2A* rearrangement	
AML with *MECOM* arrangement	
AML with in-frame bZIP *CEBPA* mutations	At least 10 % of blasts

Returning to the thought experiment: a single reptile can never be both an alligator and a crocodile simultaneously. Academia should return to basic logic, which states that a thing is always equal to itself, according to the *principle of identity*. In simpler terms, if something is “A,” then “A” is equal to “A.” Accordingly, a patient with a single disease cannot have two distinct diseases at the same time, for the simple reason that the patient has only one disease.

The purpose of classification systems is to organize and categorize objects, phenomena, information, or entities based on common characteristics, thereby facilitating their identification, study, communication, and practical application. The coexistence of the WHO and ICC systems does not make the classification of hematolymphoid tumors a simpler task, but a more complex one. The two proposals must be urgently harmonized into a single, universal classification system.

## Conflicts of interest

The authors report no conflict of interest.
